# Replication of 6 Obesity Genes in a Meta-Analysis of Genome-Wide Association Studies from Diverse Ancestries

**DOI:** 10.1371/journal.pone.0096149

**Published:** 2014-05-30

**Authors:** Li-Jun Tan, Hu Zhu, Hao He, Ke-Hao Wu, Jian Li, Xiang-Ding Chen, Ji-Gang Zhang, Hui Shen, Qing Tian, Marie Krousel-Wood, Christopher J. Papasian, Claude Bouchard, Louis Pérusse, Hong-Wen Deng

**Affiliations:** 1 Laboratory of Molecular and Statistical Genetics and Key Laboratory of Protein Chemistry and Developmental Biology of the Ministry of Education, College of Life Sciences, Hunan Normal University, Changsha, Hunan, China; 2 School of Public Health and Tropical Medicine and/or School of Medicine, Tulane University, New Orleans, Louisiana, United States of America; 3 School of Medicine, University of Missouri-Kansas City, Kansas City, Missouri, United States of America; 4 Human Genomics Laboratory, Pennington Biomedical Research Center, Baton Rouge, Louisiana, United States of America; 5 Department of Kinesiology, Laval University, Québec, Québec, Canada; Wake Forest School of Medicine, United States of America

## Abstract

Obesity is a major public health problem with a significant genetic component. Multiple DNA polymorphisms/genes have been shown to be strongly associated with obesity, typically in populations of European descent. The aim of this study was to verify the extent to which 6 confirmed obesity genes (*FTO, CTNNBL1, ADRB2, LEPR, PPARG* and *UCP2* genes) could be replicated in 8 different samples (n = 11,161) and to explore whether the same genes contribute to obesity-susceptibility in populations of different ancestries (five Caucasian, one Chinese, one African-American and one Hispanic population). GWAS-based data sets with 1000 G imputed variants were tested for association with obesity phenotypes individually in each population, and subsequently combined in a meta-analysis. Multiple variants at the *FTO* locus showed significant associations with BMI, fat mass (FM) and percentage of body fat (PBF) in meta-analysis. The strongest association was detected at rs7185735 (*P*-value = 1.01×10^−7^ for BMI, 1.80×10^−6^ for FM, and 5.29×10^−4^ for PBF). Variants at the *CTNNBL1, LEPR* and *PPARG* loci demonstrated nominal association with obesity phenotypes (meta-analysis *P*-values ranging from 1.15×10^−3^ to 4.94×10^−2^). There was no evidence of association with variants at *ADRB2* and *UCP2* genes. When stratified by sex and ethnicity, *FTO* variants showed sex-specific and ethnic-specific effects on obesity traits. Thus, it is likely that *FTO* has an important role in the sex- and ethnic-specific risk of obesity. Our data confirmed the role of *FTO, CTNNBL1, LEPR* and *PPARG* in obesity predisposition. These findings enhanced our knowledge of genetic associations between these genes and obesity-related phenotypes, and provided further justification for pursuing functional studies of these genes in the pathophysiology of obesity. Sex and ethnic differences in genetic susceptibility across populations of diverse ancestries may contribute to a more targeted prevention and customized treatment of obesity.

## Introduction

Obesity, a state in which excess lipids accumulate in various body fat depots due to a chronic imbalance between energy intake and energy expenditure, is associated with many diseases such as type 2 diabetes mellitus, hypertension, coronary heart disease, and some cancers [1]. Although the true causes of the accelerating obesity epidemic have not been fully clarified, the prevalence of obesity continues to increase around the world. If current trends continue, it is estimated that by the year 2030 nearly 50% of adults in the United States will be clinically obese [2], and the world population will include 1.12 billion obese individuals [3]. Although the impact of environmental factors is likely to be significant, many studies have shown that body weight and obesity are strongly influenced by genetic factors, with heritability estimates often in excess of 50% particularly when derived from comparisons of identical and fraternal twins [4], [5].

Most genetic studies of obesity have focused on body mass index (BMI) to evaluate whether a person is obese or not. However, BMI cannot distinguish fat mass from fat free mass [6], [7]. Alternative measurements such as percentage of body fat (PBF) and fat mass (FM) are more homogeneous and reflect body fat content more accurately than BMI [8]. Few genetic studies of obesity, however, have utilized these latter measures of body fat content.

In the past few years, extensive efforts had focused on the detection of obesity genes. In this regard, relatively few genes identified through preliminary linkage scans or candidate gene approaches have been confirmed to be truly associated with obesity by replication studies and other methodologies [9]. Among potential reasons for the failure to replicate most candidate genes or linkage peaks, one can cite small sample sizes and limited numbers of DNA variants upon which these studies were based. More recently, under the common variant–common disease hypothesis, several genome-wide association studies (GWAS) and large-scale meta-analyses of multiple GWAS on obesity (mostly using BMI as phenotype) have been conducted. Thus far, a total of 58 genetic loci, all with small effect sizes, were found to be robustly associated with obesity-related traits in multiple populations ([10]; see also: http://www.ncbi.nlm.nih.gov/gap/phegeni/). Among these genes, fat mass- and obesity-associated gene (*FTO*) stands out as the gene with the strongest significant association with obesity and it has been found to be associated with obesity in virtually all populations in which replication was attempted. Nevertheless, all genomic markers identified along with their putative genes have only been shown to have very small effects on BMI or the risk of obesity. Cumulatively, these genetic loci identified through GWAS account for less than 5% of the total heritability of BMI [11], leaving the vast majority of heritability yet unidentified. Presumably, additional variants/loci will eventually be detected with larger sample sizes combined with incorporation of rare variants, copy number variation markers, and other genomic and epigenomic features.

The leptin receptor (*LEPR*), a single-transmembrane-domain receptor of the cytokine receptor family [12], acts with an adipocyte-specific hormone leptin that regulates adipose-tissue mass through hypothalamic effects on satiety and energy metabolism. Peroxisome proliferator-activated receptor gamma(*PPARG*) is a transcription factor expressed abundantly in adipose tissue which involved in adipogenesis by activating adipocyte differentiation and mediating the expression of fat cell-specific genes [13]. Adrenoceptor beta 2, surface (*ADRB2*) is a major lipolytic receptor in human fat cells which plays a key role in regulating energy balance through both thermogenesis and lipid mobilization from adipose tissues [14]. Uncoupling protein 2 (*UCP2* **)** is an inner mitochondrial membrane transporter which dissipates the proton gradient of inner mitochondrial membranes and releases stored energy as heat, thus has an important role in energy expenditure [15]. FTO gene encoding a nucleic acid demethylase plays a role in controlling feeding behavior and energy expenditure [16]. Catenin (cadherin-associated protein), β-like 1 (*CTNNBL1*) encodes a protein homolog to β-catenin responsible for cell-to-cell adhesion and Wnt-signalling [17]. Replication of previous findings in well-designed and statistically powered studies and fine-mapping the causal variants are essential to elucidate the importance of these six genes on obesity. The present study represents a meta-analysis of samples with diverse ancestries in which we attempt to replicate associations between sequence variants in six important candidate genes of obesity and body fatness phenotypes and to explore whether the same genes contribute to obesity-susceptibility in populations of different ancestries in large study samples.

## Materials and Methods

### Study Populations

We utilized eight GWAS, four of which were “in-house” studies: (1) Quebec Family Study (**QFS**, n = 875, Caucasian Ancestry), (2) Omaha Osteoporosis Study (**OOS**; n = 998, Caucasian Ancestry), (3) Kansas-City Osteoporosis Study (**KCOS**; n = 2,283, Caucasian Ancestry) and (4) China Osteoporosis Study (**COS**; n = 1,624, Han Chinese Ancestry). Three studies(OOS, KCOS and COS) were originally designed to identify potential genes underlying osteoporosis. The other four were “external” studies deposited into the Database on Genotypes and Phenotypes (dbGaP) at the National Library of Medicine (http://www.ncbi.nlm.nih.gov/gap/): (1) Framingham Heart Study (**FHS**; n = 2,786, Caucasian Ancestry), a longitudinal and prospective cohort comprising over 16,000 Caucasian subjects spanning three generations. Based on the first two generations of the FHS families, we identified 2,786 subjects with both BMI and FM information for use in this study. (2) Indiana Fragility Study (**IFS**; n = 1,478, Caucasian Ancestry), a quantitative and cross-sectional cohort comprising premenopausal Caucasian sister pairs. (3) Women’s Health Initiative (WHI) Observational Study [18] African Sub-study (**WHI-AA**; n = 709, African Ancestry), (4) WHI Observational Study Hispanic Sub-study (**WHI-HIS**; n = 408, Hispanic Ancestry). Details regarding these studies have been published previously [19]–[21]. All studies were approved by their respective institutional ethics review boards.

### Phenotype Measurements

Several obesity-related phenotypes were measured. These include BMI, body composition (FM and PBF) measured by dual-energy X-ray absorptiometry (DXA) scanners (either Lunar Corp., Madison, WI, USA, or Hologic Inc., Bedford, MA, USA) following the manufacturer’s protocols or by underwater weighing with corrections for pulmonary residual volume. Covariates, including sex, age, age^2^, weight, height, and scanner ID (in WHI-AA and WHI-HIS), were screened with a stepwise linear regression model. Raw measurements were adjusted for significant covariates. To correct for potential population stratification, principal components (PCs) were computed and the first five PCs (i.e., PC1-PC5, explained >95% variation in each population.) derived from genome-wide genotype data were also included as covariates. Residual scores for each phenotype were normalized by inverse quantile of the standard normal distribution to impose a normal distribution on phenotypes which were then subjected to further analysis.

### Genotyping and Quality Control

All eight cohorts were genotyped using high-throughput SNP genotyping arrays (Affymetrix Inc., Santa Clara, CA; or Illumina Inc., San Diego, CA, USA) following their respective manufacturer’s protocols. Implemented in PLINK (http://pngu.mgh.harvard.edu/~purcell/plink/), quality control criteria included the following: missing data <5%, SNP call rate >95%, and Hardy-Weinberg equilibrium (HWE) P-value>1.0×10^−5^. For two family-based studies (i.e., FHS and IFS), all genotypes with Mendelian inheritance errors were set to missing. Details regarding the genotyping platforms, quality control, and data cleaning measures have been described previously [19]–[21].

### Genotype Imputation

To combine data across different genotyping platforms and to achieve a higher genome coverage, extensive genotype imputation was performed. Briefly, haplotypes of individual GWAS were first phased by a Markov Chain Haplotyping algorithm (MACH) (http://genome.sph.umich.edu/wiki/MaCH) [22], and untyped genotypes were then imputed by Minimac (http://genome.sph.umich.edu/wiki/Minimac), based on phased haplotypes, using the freely available haplotype data of the 1000 Genomes Project (as of August, 2010) as reference panels. Reference samples included 283 individuals of European ancestry, 193 individuals of Asian ancestry, and 174 individuals of African ancestry. Imputation was performed by comparing the respective panel with the closest ancestry. For each GWAS, genotypes for untyped SNPs were imputed based on relevant population’s haplotype reference panel. SNPs with *imputation quality score* (as assessed by r2.hat by Minimac) greater than 0.3 were retained in at least two studies, and with minor allele frequency (MAF) >0.05 in at least one study, were included for subsequent analyses. Prior to genotype imputation, strand orientations were checked and inconsistencies were resolved. Imputation results are summarized as an ‘allele dosage’ defined as the expected number of copies of the coded allele at that SNP (i.e., a fractional value between 0 and 2) for each genotype. In total, 2,954 genotyped or imputed autosomal SNPs spanning the *FTO*(1,275 SNPs), *ADRB2*(119 SNPs), *CTNNBL1*(483 SNPs), *LEPR*(550 SNPs), *PPARG*(435 SNPs) and *UCP2* (92 SNPs) genes were analyzed.

### Association Tests

Association was tested in each study between directly-typed or imputed SNPs and obesity phenotypes under an additive genetic model. For each study of unrelated subjects (i.e., OOS, KCOS, COS, WHI-AA, and WHI-HIS), association was examined by fitting a linear regression model using MACH2QTL (http://www.sph.umich.edu/csg/abecasis/MACH/download/), in which allele dosage was used as a predictor of phenotype. For family-based samples (i.e., QFS, FHS and IFS), a mixed linear model was used in which the effect of genetic relatedness within each pedigree was taken into consideration [23].

### Meta-analysis

Conventional meta-analysis of individual studies was performed with weights proportional to the square root of the sample size using METAL software (http://www.sph.umich.edu/csg/abecasis/metal/) [24], and *Cochran’s* Q statistic and *I* ^2^ were calculated as measures of between-study heterogeneity. Random-effect meta-analyses were performed particularly for SNPs with Q statistic P-value<0.05 or *I* ^2^ value>50%. Meta-analysis of effect size (regression slope) of the identified candidate SNP and the forest plot were performed using Review Manager (RevMan) (http://ims.cochrane.org/revman).

Regional association plots of the most significant SNPs were generated using LocusZoom [25]. Wright’s F-statistics (F_ST_) was calculated with the R package to assess genetic differences among populations using the differences in allele frequencies. We interpreted the resultant F_ST_ values based on Wright’s suggested qualitative guidelines of F_ST_ values as follow: F_ST_ = 0–0.05 indicating little population differentiation, 0.05–0.15 indicating moderate differentiation, 0.15–0.25 indicating great differentiation, and >0.25 indicating very great differentiation.

### Trans-ethnic Meta-analysis

Given the multiple ancestry groups included in the study, we further performed a trans-ethnic meta-analysis using a recent MANTRA (Meta-Analysis of Trans-ethnic Association studies) software [26] as an alternative and robust analytic approach that accounts for the trans-ethnic nature of the various cohorts. In contrast to the traditional meta-analysis, the trans-ethnic approach in MANTRA accommodates between-population heterogeneity of associated variants and their effect sizes by allowing for allelic effects to be most similar between the most closely related populations. MANTRA adopts a Bayesian framework and assumes that studies from closely related populations are more likely to share a common true effect size, and the true effect size is allowed to vary across different population clades. Default settings were used in MANTRA. Evidence in favor of association of the trait with the variant was assessed by means of a Bayes’ factor (BF).

## Results

**Figure 1:**
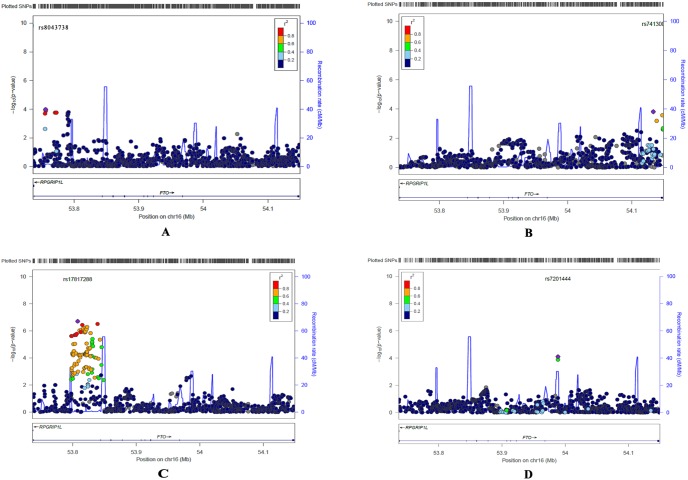
Regional plots of *FTO* gene in African (A), Chinese (B), Caucasian (C) and Hispanic populations (D), respectively. SNPs are plotted by position on the chromosome against association with BMI (−log10 P-value). Recombination rates (from HapMap) are plotted in blue to reflect the local LD structure. The SNPs surrounding the most significant SNP (in purple) are color coded to reflect their LD with this SNP (r^2^ values from the 1000 Genomes Mar 2012 AFR, ASN, EUR and AMR data, respectively).

Baseline characteristics of the subjects in each cohort are presented in Table 1. A total of 2,954 SNPs (77.2%–91.1% imputed SNPs) covering the six targeted genes were meta-analyzed. A total of 45 SNPs located in the *FTO* gene demonstrated significant associations with obesity phenotypes using a Bonfferoni corrected significance level of *P*<1.68×10^−5^. Linkage disequilibrium (LD) analysis revealed that these 45 SNPs were in almost complete LD (r^2^>0.80) and were located within the same LD block of approximately 50 kb (Figure 1). Another 125 SNPs out of the 1,275 total SNPs spanning the FTO gene in the present study showed marginal associations with obesity phenotypes (meta-analysis *P*-values ranging from 1.71×10^−5^ to 4.99×10^−2^) (Results not shown).

**Table 1:** Basic Characteristics of the Studied Samples.

Sample	SampleSize	Ancestry	Female(%)	Age	Height	Weight	BMI	FM	PBF	Measurement
				(yrs)	(m)	(kg)	(g/cm^2^)	(kg)	(%)	
QFS	875	Caucasian	57	42.4(16.8)	1.65(0.09)	75.90(21.7)	27.70(7.67)	22.60(14.32)	28.17(10.86)	Underwaterweighing
OOS	998	Caucasian	49.9	50.3(18.3)	1.71(0.10)	80.10(17.72)	27.36(5.32)	24.99(9.80)	31.48(8.72)	Hologic QDR4500W
KCOS	2283	Caucasian	75.5	51.4(13.8)	1.66(0.08)	75.16(17.47)	27.14(5.75)	24.17(10.63)	31.17(8.88)	
COS	1624	HanChinese	50.7	34.8(13.4)	1.64(0.08)	60.27(10.54)	22.21(3.02)	14.02(5.44)	23.62(8.00)	
FHS	2786	Caucasian	54.7	60.3(10.7)	1.66(0.10)	77.00(16.99)	26.95(5.08)	25.34(8.08)	36.93(9.12)	Lunar DPX-L
IFS	1478	Caucasian	100.0	32.7(7.2)	1.65(0.06)	71.66(16.90)	26.21(5.97)	25.33(12.05)	36.41(9.21)	
WHI-AA	709	African	100.0	60.9(6.9)	1.62(0.06)	83.15(17.72)	31.00(6.33)	37.53(12.65)	45.26(6.79)	
WHI-HIS	408	Hispanic	100.0	60.7(7.2)	1.57(0.06)	73.87(15.62)	28.80(5.45)	32.58(10.66)	44.73(6.90)	

Notes: Data were presented as mean (SD). Abbreviations: QFS, Quebec Family Study; OOS, Omaha osteoporosis study; KCOS, Kansas-city osteoporosis study; COS, China osteoporosis study; FHS, Framingham heart study; IFS, Indiana fragility study; WHI-AA, Women’s health initiative African American; WHI-HIS, Women’s health initiative Hispanic.

**Figure 2:**
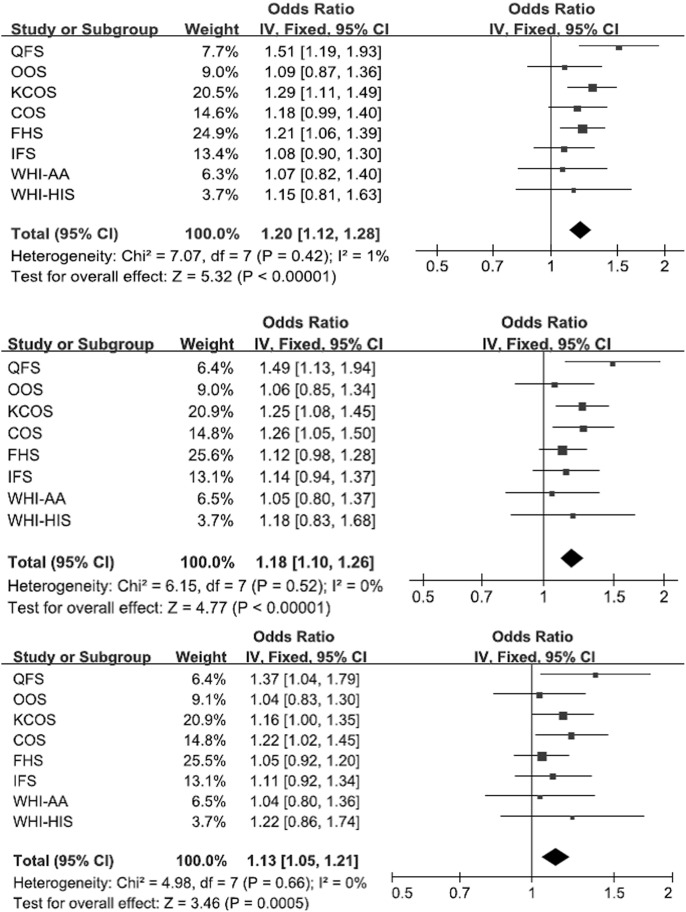
Forest plot of the association of rs7185735 and BMI (upper), FM (middle) and PBF (lower).

Table 2 shows the top 15 *FTO* SNPs associated with obesity traits. For most variants, little heterogeneity among cohorts was observed, except for rs9922708 for which moderate heterogeneity was observed (I^2^ = 43% and Q_P-value_ = 0.091). The direction of effects was consistent across studies except for three SNPs (rs9922708, rs17817449 and rs7206790). The trans-ethnic meta-analysis using MANTRA closely mirrored the results from the standard meta-analysis using METAL. The most significant association was observed for *FTO* rs7185735 (*P*-value = 1.01×10^−7^ for BMI, 1.80×10^−6^ for FM, and 5.29×10^−4^ for PBF) (Table 2), and allele-specific OR (95% CI) reached 1.20 (1.12–1.28) for BMI, 1.18(1.10–1.28) for FM and 1.13 (1.05–1.21) for PBF (Figure 2). The frequency of minor allele G at this imputed SNP ranged from 0.11 (Chinese) to 0.47 (Caucasian) in different ethnic groups. The forest plot for this SNP indicated that its strongest association was observed in QFS (OR = 1.51 for BMI, 1.49 for FM and 1.37 for PBF). Carriers of the G allele had higher BMI, FM and PBF values (Figure 2).

**Table 2:** Top 15 SNPs identified in the meta-analysis.

SNP ID	Allele	Position	Direction	BMI			FM			PBF		
				METALP value	log10BF	Posterior probability ofassociation	METALP value	log10BF	Posterior probability ofassociation	METALP value	log10BF	Posterior probability ofassociation
rs9939609	A/T	53820527	−−−−	1.13×10^−7^	4.80	0.79	1.80×10^−6^	3.30	0.56	5.29×10^−4^	1.29	0.34
rs7185735	G/A	53822651	−−−−	1.01×10^−7^	4.61	0.70	2.21×10^−6^	3.39	0.47	5.88×10^−4^	1.24	0.26
rs7202116	G/A	53821615	−−−−	1.61×10^−7^	4.59	0.70	2.11×10^−6^	3.23	0.50	5.56×10^−4^	1.25	0.27
rs7193144	C/T	53810686	++++++++	2.64×10^−7^	4.58	0.79	3.00×10^−6^	2.80	0.58	7.29×10^−4^	1.29	0.30
rs8050136	A/C	53816275	++++++++	3.16×10^−7^	4.52	0.69	2.84×10^−6^	3.05	0.46	6.42×10^−4^	1.04	0.29
rs8051591	G/A	53816752	++++++++	2.15×10^−7^	4.51	0.74	3.37×10^−6^	3.04	0.48	7.64×10^−4^	1.18	0.31
rs11075990	G/A	53819893	−−−−	2.74×10^−7^	4.49	0.74	3.43×10^−6^	3.13	0.50	6.70×10^−4^	1.21	0.29
rs3751812	T/G	53818460	++++++++	3.41×10^−7^	4.48	0.71	3.56×10^−6^	3.08	0.45	7.77×10^−4^	1.33	0.30
rs9935401	A/G	53816838	++++++++	1.81×10^−7^	4.46	0.74	3.80×10^−6^	3.07	0.52	7.14×10^−4^	1.22	0.32
rs11075989	T/C	53819877	−−−−	1.98×10^−7^	4.45	0.73	3.96×10^−6^	3.07	0.48	7.87×10^−4^	1.12	0.29
rs8043757	T/A	53813450	++++++++	3.24×10^−7^	4.42	0.76	4.92×10^−6^	3.00	0.58	9.28×10^−4^	1.28	0.29
rs17817449	G/T	53813367	−+++++++	4.97×10^−7^	4.41	0.83	4.18×10^−6^	3.00	0.68	7.35×10^−4^	1.24	0.40
rs9923233	C/G	53819198	−−−−	1.36×10^−7^	4.38	0.76	4.86×10^−6^	3.27	0.48	1.09×10^−3^	1.44	0.28
rs9936385	C/T	53819169	−−−−	2.34×10^−7^	4.38	0.73	5.51×10^−6^	3.08	0.53	8.12×10^−4^	1.27	0.33
rs17817964	T/C	53828066	++++++++	5.43×10^−7^	4.37	0.73	4.52×10^−6^	2.95	0.50	1.08×10^−3^	0.93	0.32

For the other five genes, 26 SNPs, 11 SNPs and 33 SNPs from *CTNNBL1, PPARG* and *LEPR* showed marginal significance of associations with *P*-values ranging between 1.50×10^−3^ and 4.94×10^−2^(Results not shown). The strongest associations for the *CTNNBL1, PPARG* and *LEPR* gene markers were: *CTNNBL1* rs45500793 (*P*-value = 2.68×10^−3^ for BMI, 6.34×10^−3^ for FM, and 2.06×10^−2^ for PBF), *LEPR* rs9436744 (*P*-value = 3.16×10^−3^ for BMI, 2.33×10^−2^ for FM, and 7.55×10^−2^ for PBF) and *PPARG* rs10222537 (*P*-value = 1.50×10^−3^ for BMI, 1.19×10^−2^ for FM, and 6.77×10^−2^ for PBF). We did not detect association between any variants in the *ADRB2* and *UCP2* genes and obesity phenotypes.

**Figure 3:**
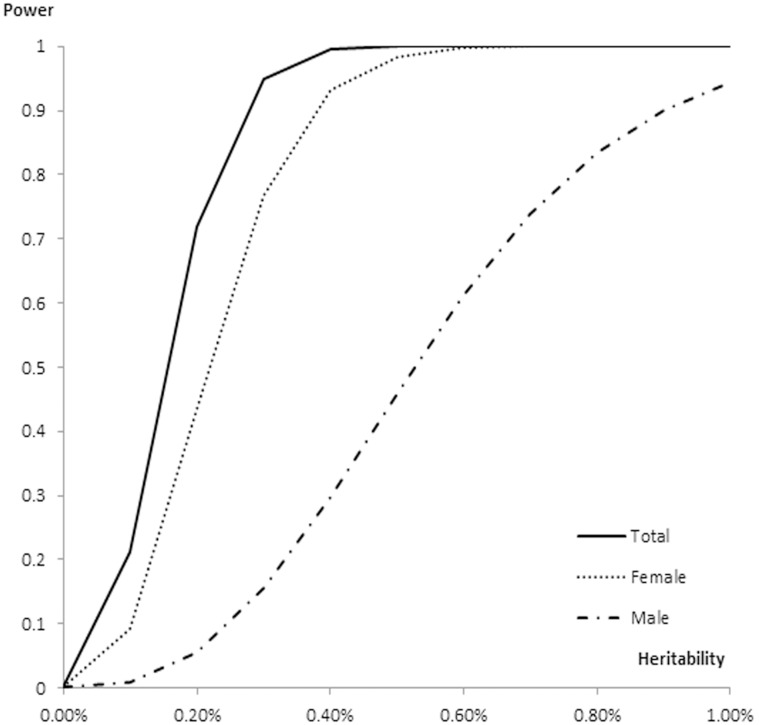
Power estimation for the total sample, females and males.

To identify potential sex-specific loci, we performed a series of meta-analyses stratified by sex. In female specific samples, 25 *FTO* SNPs were significantly associated with BMI and FM, all of which overlapped with the findings obtained with the combined sample. There was no evidence of significant associations in the male specific samples (Table 3). However, a nominally significant association was revealed at rs16952725 of *FTO* in males (*P*-value = 6.87×10^−4^ for BMI and *P*-value = 8.55×10^−3^ for FM), but not in the female specific sample (*P*-value = 0.55 for BMI and *P*-value = 0.43 for FM) (Table 3). We estimated the statistical power at various locus heritabilities for the total sample, males and females (Figure 3). Our total sample and female sample have >90% power to detect a variant explaining >0.40% of heritability. However, the male sample only have 30% power to detect a variant explaining >0.40% of heritability.

**Table 3:** P values of sex specific SNPs identified in the meta-analysis.

SNP ID	Allele	Position	Gene	BMI		FM		PBF	
				Female	Male	Female	Male	Female	Male
rs7185735	G/A	53822651	FTO	1.14×10^−6^	8.61×10^−2^	4.08×10^−4^	1.13×10^−1^	3.35×10^−3^	9.68×10^−2^
rs9939609	A/T	53820527	FTO	1.10×10^−6^	9.49×10^−2^	1.28×10^−5^	1.32×10^−1^	2.96×10^−3^	1.17×10^−1^
rs9923233	C/G	53819198	FTO	1.21×10^−6^	9.00×10^−2^	1.29×10^−5^	1.16×10^−1^	3.07×10^−3^	9.85×10^−2^
rs7202116	G/A	53821615	FTO	1.28×10^−6^	1.12×10^−1^	1.52×10^−5^	1.47×10^−1^	3.47×10^−3^	1.27×10^−1^
rs9935401	A/G	53816838	FTO	1.78×10^−6^	9.30×10^−2^	1.99×10^−5^	1.12×10^−1^	3.80×10^−3^	9.39×10^−2^
rs11075989	T/C	53819877	FTO	1.57×10^−6^	1.10×10^−1^	1.75×10^−5^	1.41×10^−1^	3.68×10^−3^	1.20×10^−1^
rs8051591	G/A	53816752	FTO	1.71×10^−6^	1.09×10^−1^	1.97×10^−5^	1.30×10^−1^	3.42×10^−3^	1.10×10^−1^
rs9936385	C/T	53819169	FTO	1.69×10^−6^	1.20×10^−1^	1.66×10^−5^	1.57×10^−1^	3.54×10^−3^	1.27×10^−1^
rs7193144	C/T	53810686	FTO	2.14×10^−6^	1.07×10^−1^	2.33×10^−5^	1.26×10^−1^	3.62×10^−3^	1.11×10^−1^
rs11075990	G/A	53819893	FTO	2.26×10^−6^	1.11×10^−1^	2.07×10^−5^	1.43×10^−1^	3.74×10^−3^	1.22×10^−1^
rs8050136	A/C	53816275	FTO	2.05×10^−6^	1.36×10^−1^	2.23×10^−5^	1.56×10^−1^	4.00×10^−3^	1.28×10^−1^
rs8043757	T/A	53813450	FTO	2.68×10^−6^	1.08×10^−1^	2.66×10^−5^	1.22×10^−1^	3.84×10^−3^	1.06×10^−1^
rs3751812	T/G	53818460	FTO	3.04×10^−6^	1.06×10^−1^	2.78×10^−5^	1.36×10^−1^	5.42×10^−3^	1.17×10^−1^
rs17817449	G/T	53813367	FTO	4.51×10^−6^	9.16×10^−2^	3.61×10^−5^	1.08×10^−1^	4.62×10^−3^	9.15×10^−2^
rs17817964	T/C	53828066	FTO	5.24×10^−6^	9.73×10^−2^	4.08×10^−5^	9.79×10^−2^	8.66×10^−3^	7.38×10^−2^
rs16952725	C/G	54014267	FTO	5.52×10^−1^	6.87×10^−4^	4.32×10^−1^	8.56×10^−3^	3.49×10^−1^	1.91×10^−2^

Finally, we examined potential differences of SNP association with obesity phenotypes among the four ethnic groups (Chinese, Caucasians, African Americans and Hispanic-Americans). In Caucasians, we found that 35 *FTO* SNPs were significantly associated with BMI and FM (*P*<1.68×10^−5^); 28 of these 35 SNPs overlapped with those derived from the analysis performed on the combined samples. The most significant association was observed for rs17817288 in *FTO* (*P*-value = 1.96×10^−7^ for BMI, 2.76×10^−5^ for FM, and 2.39×10^−3^ for PBF). However, these significant *FTO* SNPs in Caucasians showed no or weak evidence of association in non-Caucasian populations (Table 4, Figure 1). Therefore, the association results of FTO obtained with the combined samples were mostly contributed by the Caucasian group. For example, P-value was 1.01×10^−7^ for the most significant SNP rs7185735 in the combined samples for BMI, 4.90×10^−7^ in Caucasians, 7.47×10^−2^ in Chinese, 6.05×10^−1^ in African-Americans and 4.45×10^−1^ in Hispanic-Americans. In non-Caucasian populations, we only found marginally significant associations. Two *FTO* SNPs (rs7201444 and rs13335146) specific in Hispanic-Americans, 14 *FTO* SNPs and one *LEPR* SNP(rs9436299) specific in African Americans and one *CTNNBL1* SNP (rs45500793) specific in Chinese showed marginally significant associations (*P*-value<1×10^−3^) (Table 4, Figure 1). Table 4 listed 2 SNPs specific in Hispanic-Americans, 15 SNPs specific in African Americans, 1 SNP specific in Chinese and top 15 SNPs specific in Caucasians. To assess genetic differences for these ethnic-specific SNPs (listed in Table 4) among the eight populations, F_ST_ were calculated. Nine *FTO* variants (rs1861869, rs7186521, rs17817288, rs8044769, rs11075987, rs9935401, rs8051591, rs7193144, rs8043757) and *CTNNBL1* SNP (rs45500793) showed little population differentiation (F^ST^ = 0–0.05). F_ST_ of other SNPs ranged from 0.05 to 0.31, suggesting moderate/great genetic differentiation. FTO rs7201444 showed very great differentiation with the frequency of minor allele A ranging from 0.001 (Chinese) to 0.365 (African-Americans).

**Table 4:** P values of ethnic-specific SNPs identified in the meta-analysis.

SNP ID	Allele	Gene	Position	BMI				FM				PBF				Fst
				CHI6	AFR	His	CEU	CHI6	AFR	His	CEU	CHI6	AFR	His	CEU	
rs7201444^a^	C/A	FTO	53988511	5.03×10^−1^	5.83×10^−1^	7.83×10^−5^	1.51×10^−1^	3.74×10^−1^	6.04×10^−1^	2.97×10^−5^	9.66×10^−2^	3.04×10^−1^	9.93×10^−1^	1.66×10^−4^	1.42×10^−1^	0.31
rs13335146^a^	G/T	FTO	53988493	2.61×10^−1^	6.13×10^−1^	1.29×10^−4^	1.22×10^−1^	2.34×10^−1^	7.43×10^−1^	5.41×10^−5^	9.18×10^−2^	2.36×10^−1^	6.06×10^−1^	2.61×10^−4^	1.38×10^−1^	0.20
rs8043738^b^	T/C	FTO	53756033	5.64×10^−1^	1.09×10^−4^	5.06×10^−1^	7.33×10^−2^	9.07×10^−1^	2.83×10^−4^	4.27×10^−1^	4.05×10^−2^	9.57×10^−1^	1.49×10^−3^	4.55×10^−1^	9.09×10^−2^	0.11
rs8063472^b^	C/T	FTO	53756133	3.32×10^−1^	1.33×10^−4^	5.98×10^−1^	4.82×10^−2^	6.50×10^−1^	3.26×10^−4^	5.34×10^−1^	3.02×10^−1^	8.43×10^−1^	1.94×10^−3^	5.53×10^−1^	7.90×10^−2^	0.10
rs8058460^b^	T/C	FTO	53756137	3.31×10^−1^	1.33×10^−4^	5.97×10^−1^	4.82×10^−2^	6.50×10^−1^	3.27×10^−4^	5.33×10^−1^	3.02×10^−2^	8.43×10^−1^	1.94×10^−3^	5.52×10^−1^	7.89×10^−2^	0.10
rs1077129^b^	A/G	FTO	53791411	5.39×10^−1^	1.69×10^−4^	4.38×10^−1^	1.63×10^−1^	8.36×10^−1^	6.61×10^−4^	5.32×10^−1^	4.91×10^−1^	8.21×10^−1^	1.70×10^−2^	5.87×10^−1^	8.20×10^−1^	0.05
rs8059991^b^*	G/A	FTO	53772346	5.53×10^−1^	1.78×10^−4^	4.56×10^−1^	1.01×10^−2^	9.44×10^−1^	4.37×10^−4^	3.86×10^−1^	5.85×10^−3^	8.83×10^−1^	2.80×10^−3^	4.11×10^−1^	2.23×10^−2^	0.10
rs8048396^b^	A/C	FTO	53770749	7.71×10^−1^	1.78×10^−4^	4.66×10^−1^	6.99×10^−2^	7.85×10^−1^	4.21×10^−4^	3.95×10^−1^	4.04×10^−2^	6.53×10^−1^	2.53×10^−3^	4.19×10^−1^	8.70×10^−2^	0.11
rs6499641^b^*	A/T	FTO	53772626	7.07×10^−1^	1.83×10^−4^	4.56×10^−1^	1.00×10^−2^	9.44×10^−1^	4.46×10^−4^	3.86×10^−1^	5.82×10^−3^	8.23×10^−1^	2.85×10^−3^	4.10×10^−1^	2.23×10^−2^	0.10
rs1861869^b^	C/G	FTO	53790181	4.36×10^−1^	1.91×10^−4^	7.32×10^−1^	3.09×10^−2^	9.46×10^−1^	2.11×10^−4^	9.11×10^−1^	1.43×10^−1^	9.84×10^−1^	2.15×10^−3^	9.46×10^−1^	4.17×10^−1^	0.04
rs7205986^b^*	G/A	FTO	53755146	5.36×10^−1^	2.15×10^−4^	5.30×10^−1^	1.11×10^−2^	8.46×10^−1^	5.19×10^−4^	4.51×10^−1^	6.44×10^−3^	9.78×10^−1^	3.27×10^−3^	4.77×10^−1^	2.40×10^−2^	0.10
rs9436299^b^	A/C	LEPR	65892888	7.40×10^−2^	2.71×10^−3^	7.56×10^−1^	1.57×10^−1^	4.84×10^−1^	7.60×10^−4^	6.05×10^−1^	3.44×10^−1^	9.47×10^−1^	2.01×10^−3^	2.33×10^−1^	5.66×10^−1^	0.07
rs17217144^b^	T/C	FTO	53790762	5.40×10^−1^	2.32×10^−4^	4.41×10^−1^	1.70×10^−1^	8.40×10^−1^	7.89×10^−4^	5.45×10^−1^	5.02×10^−1^	8.25×10^−1^	1.60×10^−2^	6.02×10^−1^	8.27×10^−1^	0.05
rs2892469^b^	T/C	FTO	53789999	5.26×10^−1^	2.79×10^−4^	5.01×10^−1^	1.54×10^−1^	8.53×10^−1^	1.02×10^−3^	6.06×10^−1^	4.54×10^−1^	8.34×10^−1^	2.25×10^−2^	6.57×10^−1^	7.83×10^−1^	0.05
rs1861868^b^	T/C	FTO	53790402	5.48×10^−1^	5.28×10^−4^	4.91×10^−1^	6.28×10^−2^	8.42×10^−1^	1.29×10^−3^	6.14×10^−1^	2.42×10^−1^	8.36×10^−1^	1.61×10^−2^	6.18×10^−1^	5.17×10^−1^	0.07
rs7186521^b^	G/A	FTO	53792922	9.33×10^−1^	7.11×10^−4^	4.20×10^−1^	7.68×10^−2^	6.69×10^−1^	1.80×10^−3^	5.02×10^−1^	2.70×10^−1^	8.71×10^−1^	2.26×10^−2^	5.54×10^−1^	5.66×10^−1^	0.03
rs7184874^b^	T/C	FTO	53792439	5.50×10^−1^	7.99×10^−4^	4.46×10^−1^	5.66×10^−2^	8.32×10^−1^	1.49×10^−3^	5.62×10^−1^	2.33×10^−1^	8.29×10^−1^	1.32×10^−2^	5.94×10^−1^	5.28×10^−1^	0.07
rs45500793^c^	T/G	CTNNNBL1	36488285	1.10×10^−2^	NA	1.40×10^−2^	1.03×10^−1^	1.52×10^−3^	NA	4.35×10^−2^	2.59×10^−1^	6.00×10^−4^	NA	2.24×10^−1^	4.66×10^−1^	0.01
rs17817288^d^*	A/G	FTO	53807764	9.52×10^−1^	4.64×10^−1^	7.10×10^−1^	1.96×10^−7^	7.34×10^−1^	6.67×10^−1^	5.49×10^−1^	2.76×10^−5^	7.38×10^−1^	9.74×10^−1^	7.82×10^−1^	2.39×10^−3^	0.01
rs8044769^d^*	T/C	FTO	53839135	8.22×10^−1^	8.65×10^−1^	8.39×10^−1^	2.99×10^−7^	7.89×10^−1^	9.13×10^−1^	9.77×10^−1^	3.65×10^−5^	9.52×10^−1^	7.37×10^−1^	7.51×10^−1^	6.95×10^−3^	0.03
rs11075987^d^*	T/G	FTO	53815161	6.77×10^−1^	3.62×10^−1^	6.88×10^−1^	3.54×10^−7^	5.05×10^−1^	4.97×10^−1^	8.00×10^−1^	4.92×10^−5^	5.77×10^−1^	5.18×10^−1^	5.99×10^−1^	6.56×10^−3^	0.04
rs7185735^d^*	G/A	FTO	53822651	7.47×10^−2^	6.05×10^−1^	4.45×10^−1^	4.90×10^−7^	1.14×10^−2^	7.11×10^−1^	3.52×10^−1^	4.66×10^−5^	3.03×10^−2^	7.67×10^−1^	2.67×10^−1^	6.84×10^−3^	0.05
rs9939609^d^*	A/T	FTO	53820527	7.64×10^−2^	5.82×10^−1^	4.44×10^−1^	5.68×10^−7^	1.18×10^−2^	6.81×10^−1^	3.36×10^−1^	6.03×10^−5^	3.23×10^−2^	7.38×10^−1^	3.43×10^−1^	7.84×10^−3^	0.08
rs9935401^d^*	A/G	FTO	53816838	7.81×10^−2^	7.00×10^−1^	4.91×10^−1^	6.69×10^−7^	9.63×10^−3^	9.32×10^−1^	3.60×10^−1^	5.66×10^−5^	2.53×10^−2^	9.65×10^−1^	2.47×10^−1^	7.44×10^−3^	0.04
rs7202116^d^*	G/A	FTO	53821615	7.77×10^−2^	6.31×10^−1^	4.38×10^−1^	7.54×10^−7^	1.22×10^−2^	7.36×10^−2^	3.34×10^−1^	7.35×10^−5^	3.30×10^−2^	7.89×10^−1^	2.44×10^−1^	8.91×10^−3^	0.05
rs8051591^d^*	G/A	FTO	53816752	7.82×10^−2^	6.83×10^−1^	4.97×10^−1^	8.24×10^−7^	9.58×10^−3^	8.93×10^−2^	3.64×10^−1^	7.22×10^−5^	2.52×10^−2^	8.68×10^−1^	2.48×10^−1^	8.63×10^−3^	0.04
rs17817449^d^*	G/T	FTO	53813367	8.09×10^−2^	8.74×10^−1^	5.14×10^−1^	8.46×10^−7^	1.05×10^−2^	7.65×10^−1^	3.75×10^−1^	6.32×10^−5^	2.79×10^−2^	8.42×10^−1^	2.50×10^−1^	7.11×10^−3^	0.07
rs9923233^d^*	C/G	FTO	53819198	5.94×10^−2^	5.69×10^−1^	4.87×10^−1^	8.55×10^−7^	7.57×10^−2^	6.68×10^−1^	3.59×10^−1^	7.53×10^−5^	2.18×10^−2^	7.25×10^−1^	2.47×10^−1^	9.17×10^−3^	0.05
rs7193144^d^*	C/T	FTO	53810686	7.86×10^−2^	7.05×10^−1^	5.31×10^−1^	9.26×10^−7^	9.62×10^−3^	8.85×10^−1^	3.84×10^−1^	7.81×10^−5^	2.53×10^−2^	8.84×10^−1^	2.53×10^−1^	8.91×10^−3^	0.04
rs3751812^d^*	T/G	FTO	53818460	7.57×10^−2^	8.85×10^−1^	4.92×10^−1^	9.71×10^−7^	1.04×10^−2^	9.56×10^−1^	3.62×10^−1^	8.89×10^−5^	2.78×10^−2^	8.59×10^−1^	2.47×10^−1^	9.68×10^−3^	0.10
rs11075990^d^*	G/A	FTO	53819893	7.52×10^−2^	7.22×10^−1^	4.71×10^−1^	1.08×10^−6^	1.15×10^−2^	7.39×10^−1^	3.51×10^−1^	9.69×10^−5^	3.14×10^−2^	7.12×10^−1^	2.47×10^−1^	1.07×10^−2^	0.06
rs8050136^d^*	A/C	FTO	53816275	7.82×10^−2^	7.34×10^−1^	4.99×10^−1^	1.13×10^−6^	9.53×10^−3^	9.74×10^−1^	3.66×10^−1^	9.16×10^−5^	2.50×10^−2^	9.92×10^−1^	2.48×10^−1^	1.01×10^−2^	0.07
rs8043757^d^*	T/A	FTO	53813450	7.84×10^−2^	7.22×10^−1^	5.21×10^−1^	1.15×10^−6^	9.58×10^−3^	8.97×10^−1^	3.79×10^−1^	8.53×10^−5^	2.52×10^−2^	8.86×10^−1^	2.53×10^−1^	9.15×10^−3^	0.04

Notes: ^a^ SNPs specific in Hispanic-Americans, ^b^ SNPs specific in African Americans, ^c^ SNP specific in Chinese, ^d^ top 15 SNPs specific in Caucasians, and *SNPs previously reported.

In addition, we examined the association of previously reported SNPs of the six genes among our dataset (Table 5). All of the 10 previously reported *FTO* SNPs (rs1421085, rs1558902, rs17817449, rs9941349, rs8050136, rs1558902, rs1121980, rs7202116, rs9939609 and rs9930506) showed significant/nominal associations with BMI, FM and/or PBF. Two *PPARG* SNPs (rs1801282 and rs3856806) and 3 *CTNNBL1* variants (rs6013029, rs16986921 and rs6020712) showed nominal associations with obesity traits.

**Table 5:** Comparisons of association results of previous reported loci with the current study.

SNPs previously detected						Present meta-analysis P-value		
SNP ID	Allele	REF	P-value	Position	gene	BMI	FM	PBF
rs1421085	C/T	[58]	1.00×10^−28^	53800954	FTO	3.97×10^−6^	2.06×10^−5^	1.38×10^−3^
rs1558902	A/T	[59]	7.00×10^−13^	53803574	FTO	3.50×10^−6^	2.09×10^−5^	1.72×10^−3^
rs17817449	G/T	[60]	2.00×10^−12^	53813367	FTO	4.97×10^−7^	5.51×10^−6^	8.12×10^−4^
rs9941349	T/C	[61]	6.00×10^−12^	53825488	FTO	1.86×10^−5^	7.29×10^−5^	4.83×10^−3^
rs8050136	A/C	[62]	4.00×10^−8^	53816275	FTO	3.16×10^−7^	4.92×10^−6^	9.28×10^−4^
rs1558902	A/T	[41]	1.00×10^−7^	53803574	FTO	3.50×10^−6^	2.09×10^−5^	1.72×10^−3^
rs1121980	A/G	[63]	1.00×10^−7^	53809247	FTO	4.87×10^−5^	2.37×10^−4^	7.72×10^−3^
rs7202116	G/A	[64]	2.4×10^−10^	53821615	FTO	1.61×10^−7^	3.00×10^−6^	7.29×10^−4^
rs9939609	A/T	[65]	3×10^−35^	53820527	FTO	1.13×10^−7^	2.21×10^−6^	5.88×10^−4^
rs9930506	G/A	[38]	8.6×10^−7^	53830465	FTO	2.13×10^−5^	6.07×10^−5^	4.30×10^−3^
rs659366	T/C	[32]	P<0.05	73694754	UCP2	3.97×10^−6^	2.06×10^−5^	1.38×10^−3^
rs660339	A/G	[32]	P<0.05	73689104	UCP2	3.50×10^−6^	2.09×10^−5^	1.72×10^−3^
rs6013029	T/G	[17]	2.69×10^−7^	36399580	CTNNBL1	1.90×10^−2^	1.40×10^−2^	2.00×10^−2^
rs16986921	T/C	[17]	5.88×10^−7^	36382521	CTNNBL1	2.00×10^−2^	1.40×10^−2^	2.00×10^−2^
rs6020712	A/G	[17]	7.92×10^−7^	36386612	CTNNBL1	2.00×10^−2^	1.40×10^−2^	1.70×10^−2^
rs6020846	G/A	[17]	2.45×10^−5^	36405667	CTNNBL1	9.8×10^−2^	6.4×10^−2^	8.40×10^−2^
rs1801282	G/C	[34]	P<0.05	12393125	PPARG	2.00×10^−1^	1.80×10^−2^	4.60×10^−3^
rs3856806	T/C	[34]	P<0.05	12475557	PPARG	4.10×10^−1^	1.00×10^−1^	4.00×10^−2^
rs1137101	A/G	[35]	P<0.05	66058513	LEPR	6.60×10^−1^	7.40×10^−1^	5.80×10^−1^
rs1137100	A/G	[35]	P<0.05	66036441	LEPR	7.60×10^−1^	7.90×10^−1^	3.00×10^−1^
rs1042713	A/G	[33]	P<0.05	148206440	ADRB2	9.90×10^−1^	0.25×10^−1^	1.90×10^−1^
*rs*1042714	G/C	[33]	P<0.05	148206473	ADRB2	4.40×10^−1^	7.80×10^−1^	7.70×10^−1^

## Discussion

In this study we investigated the associations between 6 genes and obesity phenotypes, including direct measures of body fatness, using a dense set of variants genotyped or imputed in 11,161 subjects from four different ethnic backgrounds (Chinese, Caucasian, African-Americans and Hispanic-Americans). Our findings in this multiethnic population confirmed the importance of the *FTO* gene for obesity risk in humans. A total of 45 SNPs located in the *FTO* gene showed significant associations with the obesity phenotypes. The *FTO* protein affects demethylation of nuclear RNA in vitro [27], but whether the efficiency of this process depends on the *FTO* genotype or how this may be related to the observed effects on BMI or body fatness is not clear.

At present, the strongest associations between *FTO* SNPs and BMI belong to intronic SNPs, which might have a role in the regulation of *FTO* and/or nearby genes. It is critical to recognize, however, that associated SNPs are not necessarily causal SNPs underlying the association, and that the functional variants are still unknown. A recent study reported that rs7202116 G allele creates a CpG site along with other variants in perfect linkage disequilibrium with it [28], and these risk alleles may potentially have increased DNA methylation. Bioinformatic analyses also revealed that six *FTO* SNPs (rs11642015, rs17817497, rs3751812, rs17817964, rs62033408, and rs1421085) were located within candidate intronic regulatory elements and that two SNPs (rs11642015 and rs1421085) were predicted to have allele-specific binding affinities for different transcription factors [29]. Specifically, the T allele at rs11642015 binds Paired box protein 5 (PAX5) while the C allele at rs1421085 is predicted to have a substantially reduced binding affinity for Cut-like homeobox 1 (CUX1). Further investigation is warranted to identify potentially functional SNPs and the mechanisms by which various alleles at *FTO* influence the level of adiposity.

We detected moderate associations for multiple variants from the *CTNNBL1*, *PPARG* and *LEPR* genes, but did not detect association with any variants in *ADRB2* and *UCP2*. Our data confirmed the associations with adiposity for three previously reported *CTNNBL1* variants (rs6013029, rs16986921 and rs6020712) [17]. This finding is consistent with the results from Andreasen and co-workers, who in a study comprising 18,014 Danish participants found that the *CTNNBL1* rs6013029 T-allele and the rs6020846 G-allele confer an increased risk of developing obesity, especially morbid obesity [30]. Interestingly, a German population-based study (KORA) [31] failed to demonstrate association of *CTNNBL1* variant rs6013029 with obesity or BMI. The discrepancy might be due to population-specific differences and/or insufficient statistical power. For the most frequently studied coding variants of *UCP2* [32], *ADRB2* [33], *PPARG* [34] and *LEPR* [35] genes, only rs1801282 and rs3856806 in *PPARG* gene showed nominal associations with BMI and/or PBF.

There are substantial ethnic differences in the prevalence of excess body weight and obesity. Although differences in lifestyle are likely to account for some of the observed differences, genetic variability could also play a major role. Among the four ethnic groups (Chinese, Caucasian, African-Americans and Hispanic populations), we found that 35 SNPs in intron 1 of *FTO* gene were significantly associated with indicators of obesity in Caucasians. All of the SNPs overlapped with those reported in a previous meta-analysis study [36]. There was very limited or no evidence for associations between these SNPs and adiposity in the other ethnic groups. On the other hand, two *FTO* SNPs (rs7201444 and rs13335146) in intron 8 specific in Hispanic Americans, 11 *FTO* SNPs (rs8043738, rs8063472, rs8058460, rs1077129, rs8048396, rs1861869, rs17217144, rs2892469, rs1861868, rs7186521, rs7184874) in intron 1 specific in African Americans showed moderate evidence for associations that were not reported by previous studies. While several previous studies reported association between *FTO* SNPs and obesity-related phenotypes in Hispanic Americans [37]–[39], African Americans [29], [40] and Asian populations [41]–[44], these studies were relatively small in sample sizes and showed mixed results. Wing et al. [39] genotyped 26 SNPs in intron 1 of *FTO* in 373 Hispanic Americans and observed associations between BMI and several SNPs that were previously reported to be associated with obesity (rs9939609, rs8050136, rs1121980, rs1421085, rs17817449 and rs3751812), and four other SNPs (rs8047395, rs10852521, rs8057044 and rs8044769). Song et al. [37] and Scuteri et al. [38] replicated associations of BMI with the well-known rs9939609 [37] and rs9930506 [38] in Hispanic Americans. However, their findings were not confirmed in our Hispanic Americans. The two Hispanic-American specific SNPs found in our study were in intron 8 of *FTO*. Interestingly, a detailed mapping (262 tag SNPs across the entire *FTO* gene) in individuals of African American descent demonstrated significant association for seven SNPs (rs708262, rs11076017, rs16952725, rs9932411, rs7191513, rs2689269, rs16952987) in intron 8 [40]. Our study together with Adeyemo et al. suggests the intron 8 of FTO is a second site, in addition to intron 1, playing a role in the association between FTO and indicators of obesity.

Previous studies in African Americans showed either very limited or no evidence for associations with the SNPs initially reported in European populations, such as rs9939609, rs1121980, rs17817449 and rs8050136 [29]. More comprehensive evaluations of the *FTO* locus identified several other variants, rs56137030 [29], rs3751812 [45], [46], rs1108102 [39] and rs8057044 [47] that showed associations with BMI in African Americans. Among these variants, only rs1108102 (*P*-value = 6.15×10^−3^) replicated in our African American sample. Among the 14 *FTO* SNPs specific to African Americans, rs1861868 was previously found to be the strongest SNP associated with BMI in Old Order Amish individuals with low physical activity (P<0.001) [48]. rs1861869 and rs7186521 were found to be associated with weight and waist circumference in 843 unrelated individuals from an island population in the eastern Adriatic coast of Croatia [49]. The allele frequencies for most of the SNPs indicated moderate population differentiation, and thus could potentially lead to variable genetic impact on obesity phenotypes across populations. In summary, our study replicated the associations of *FTO* intron 1 variants with BMI, FM and PBF in Caucasians, and confirmed the importance of variants in intron 8 as well. Therefore, our results suggest that there are ethnic differences with regard to the effects of *FTO* on obesity and body fatness.

Marked sex differences in the prevalence of obesity between women and men suggest sex-specific genetic impacts on obesity risk, even though lifestyle and dietary factors are also likely to contribute to these differences. Furthermore, genetic factors may interact with levels of physical activity to modify obesity risk [50]. To determine if the effect of genetic variants differed by sex, we stratified by sex and found that 25 *FTO* SNPs were specifically associated with obesity in females only. This is consistent with a few studies that have shown sex-differences in the heritability of BMI and fat percentage [51], [52]. In contrast, a nominally significant association was revealed at rs16952725 of the *FTO* gene in males only, but not in females. Our findings are further supported by a recent study in children which found that the *FTO* variant rs9939609 showed association with obesity and BMI among girls but not among boys [53]. A recent genome-wide association study modeling the effect of genotype-by-sex interaction on obesity phenotypes demonstrated sex-influenced associations between genetic variation at the *LYPLAL1* locus and obesity-related traits [54]. This study suggests that *FTO* may be a gene playing a role in the commonly observed sex-dimorphism in adiposity.

The majority of obesity loci have been discovered through GWAS in individuals of European descent, and, more recently, in Asians as well. Okada et al. performed a GWAS with BMI in 62,245 East Asian subjects and observed a significant association with *FTO* rs12149832 (*P*-value = 4.8×10^−22^) [55]. However, our study failed to replicate the association of *FTO* rs12149832. Several other studies replicated *FTO* rs9939609 [41]–[43], [56], [57] and rs8050136 [44] with obesity and BMI in Chinese or Asians. Our study showed weak evidence of association with FM and PBF for rs9939609 and rs8050136 (*P*-values range from 9.53×10^−3^ to 3.23×10^−2^), but not with BMI. Lu and Loos reviewed the transferability of 36 GWAS identified BMI-associated SNPs between European and East Asian ancestry populations using both SNP-to-SNP and locus-wide comparisons [32]. SNP-to-SNP comparisons suggest that of the 32 SNPs found in European populations, 8 SNPs were non-polymorphic and another 6 showed no convincing evidence of association in East Asians and two of the four loci identified in East Asians showed some evidence of transferability to European populations [32]. However, locus-wide analyses suggested the more extensive transferability. For example, *CDKAL1* rs9356744 showed genome-wide significant association with BMI in East Asians but not in European populations (*P*-value = 0.19). When examining the locus surrounding rs9356744, however, other SNPs that are not in LD with rs9356744 were actually associated with BMI in European populations (*P*-values<10^−3^). Replication studies in other populations that scan the entire gene (rather than just one or a few SNPs) may lead to the discovery of other important genetic variants. Studying populations of different ancestries (especially those with smaller LD) could help fine-map disease or trait loci, eventually pinpointing the causal variant (s) and gene(s). Our study used a comprehensive approach by thoroughly examining six genes with dense SNP coverage to search for population-specific and/or shared obesity loci in multiple ancestry groups.

An important limitation of the current study is the relatively small numbers of male subjects (29% of total subjects) and subjects from the three non-Caucasian ethnic groups (Chinese, African and Hispanic populations, 25% of the total subjects). These low numbers may have contributed to the nominal associations detected in these sub-groups. Nevertheless, the present study provided additional evidence supporting the presence of ethnic- and sex-differences for some prominent obesity variants and perhaps genes.

In conclusion, we have attempted to replicate previously reported associations between multiple DNA common variants relating to 6 obesity genes in populations representing four ethnicities. We were able to find confirmatory evidence for contributions of *FTO*, *CTNNBL1*, *LEPR and PPARG* related genomic variants to human variation in adiposity. In particular, *FTO* variants showed sex-specific and ethnic-specific associations with adiposity traits.
